# Data set of in silico simulation for the production of clavulanic acid and cephamycin C by *Streptomyces clavuligerus* using a genome scale metabolic model

**DOI:** 10.1016/j.dib.2019.103992

**Published:** 2019-05-15

**Authors:** Stephania Gómez-Cerón, David Galindo-Betancur, Howard Ramírez-Malule

**Affiliations:** Universidad del Valle, Escuela de Ingeniería Química, A.A. 25360 Cali, Colombia

## Abstract

*Streptomyces clavuligerus* (*S. clavuligerus*) is a Gram-positive bacterium which produced clavulanic acid (CA) and cephamycin C (CephC). In this data article, a curated genome scale metabolic model of *S. clavuligerus* is presented. A total of eighteen objective functions were evaluated for a better representation of CA and CephC production by *S. clavuligerus*. The different objective functions were evaluated varying the weighting factors of CA and CephC between 0, 1 y 2, whereas for the case of biomass the weight factor was varied between 1 and 2. A robustness analysis, by mean of flux balance analysis, showed five different metabolic phenotypes of *S. clavuligerus* as a function of oxygen uptake: (I) and (II) biomass production, (III) biomass and CephC production, (IV) simultaneous production of biomass, CA and CephC and (V) production of biomass and CA. Data of shadow prices and reduced cost are also presented.

**Table:** Specifications Table

Subject area	*Modelling and Simulation, Biotechnology*
More specific subject area	*Flux balance analysis*
Type of data	*Table, equation, figure*
How data was acquired	*Software COBRA Toolbox v3.0 running in a Matlab® environment, using Gurobi optimization software.*
Data format	*Simulated*
Experimental factors	*A total of eighteen objective functions were evaluated varying the weighting factors of CA and cephamycin C between 0, 1 y 2, whereas for the case of biomass the weight factor was varied between 1 and 2.*
Experimental features	*Diverse metabolic phenotypes for the production of CA and cephamycin C by Streptomyces clavuligerus, through a linear combination of the weighting factor on the objective function, were evaluated.*
Data source location	*Universidad del Valle, Escuela de Ingeniería Química, A.A. 25360 Cali, Colombia.*
Data accessibility	*Data is presented in this article only.*
Related research article	*H. Ramirez-Malule, S. Junne, M.N. Cruz-Bournazou, P. Neubauer, R. Ríos-Estepa, Streptomyces clavuligerus shows a strong association between TCA cycle intermediate accumulation and clavulanic acid biosynthesis, Appl. Microbiol. Biotechnol. 102 (2018) 4009–4023.*

**Table:** 

**Value of the data** • An updated genome scale metabolic model of *Streptomyces clavuligerus* is presented. • The data will be useful for the understanding the metabolic phenotypes during the simultaneous production of clavulanic acid and cephamycin C by *Streptomyces clavuligerus*. • This data will be useful to the researchers and scientific community working on clavulanic acid and cephamycin C production.

## 1 Data

A total of twenty-four reactions were added for a better representation of the production of clavulanic acid (CA) and cephamycin C (CephC) by *Streptomyces clavuligerus* (see Table 1).

**Table 1:** Added/removed reactions on the genome scale metabolic network of *S. clavuligerus* reported by Ramirez-Malule et al. (2018).

Reaction	Comment	Reference
lys_L[c] <=> 15dap[c] + co2[c]	Intracellular reaction/Added	[1]
xyl_D[c] <=> xylu_D[c]	Intracellular reaction/Added	[2]
tre[c] + h2o[c] <=> 2 glc_D[c]	Intracellular reaction/Added	[3]
atp[c] + Dall[c] <=> adp[c] + all6p[c]	Intracellular reaction/Added	[4]
galur[c] <=> dtgt[c]	Intracellular reaction/Added	[5]
tsul[c] + cn[c] <=> so3[c] + tcynt[c]	Intracellular reaction/Added	[6]
xil[c] + nadp[c] <=> xylu_L[c] + nadph[c] + h[c]	Intracellular reaction/Added	[7]
acser[c] + tsul[c] <=> sucys[c] + ac[c]	Intracellular reaction/Added	[8]
xylu_L[c] <=> lyx_L[c]	Intracellular reaction/Added	[9]
mndl[c] <=> cyan[c] + bzal[c]	Intracellular reaction/Added	[9]
digalur[c] + h2o[c] <=> 2 galur[c]	Intracellular reaction/Added	[9]
LalaDglu[c] <=> LalaLglu[c]	Intracellular reaction/Removed	–
dtgt[e] <=> dtgt[c]	Transport reaction/Added	–
Dall[e] <=> Dall[c]	Transport reaction/Added	–
mndl[e] <=> mndl[c]	Transport reaction/Added	–
cn[e] <=> cn[c]	Transport reaction/Added	–
sucys[e] <=> sucys[c]	Transport reaction/Added	–
digalur[e] <=> digalur[c]	Transport reaction/Added	–
xil[e] <=> xil[c]	Transport reaction/Added	–
dtgt[e] →	Exchange reaction/Added	–
Dall[e] →	Exchange reaction/Added	–
mndl[e] →	Exchange reaction/Added	–
cn[e] <=>	Exchange reaction/Added	–
sucys[e] →	Exchange reaction/Added	–
digalur[e] →	Exchange reaction/Added	–
xil[e] →	Exchange reaction/Added	–

An array of eighteen combinations of different objectives functions varying the weighting factor of the slack variables was evaluated (see Table 2). The objective function was the maximization of biomass, CA and CephC. In order to evaluated the functionally of the objective functions the weighting factor of biomass, CA and CephC were varied (see experimental design). Table 2 also shows the metabolic scenarios where CA and CephC are produced or not.

**Table 2:** Relative weighting vector used to generate all the objective functions evaluated.

No. Objective function	Weighting factors			Robustness analysis: oxygen		
	Biomass	Clavulanic acid	Cephamycin C	Biomass	Clavulanic acid	Cephamycin C
1	1	0	0	YES	NO	NO
2	1	0	1	YES	NO	NO
3	1	0	2	YES	NO	YES
4	1	1	0	YES	YES	NO
5	1	1	1	YES	YES	NO
6	1	1	2	YES	YES	YES
7	1	2	0	YES	YES	NO
8	1	2	1	YES	YES	NO
9	1	2	2	YES	YES	NO
10	2	0	0	YES	NO	NO
11	2	0	1	YES	NO	NO
12	2	0	2	YES	NO	NO
13	2	1	0	YES	YES	NO
14	2	1	1	YES	YES	NO
15	2	1	2	YES	YES	NO
16	2	2	0	YES	YES	NO
17	2	2	1	YES	YES	NO
18	2	2	2	YES	YES	NO

The objective function No. 6 was the only one that included a metabolic phenotype that produced CA and CephC, simultaneously. Table 3 shows the fluxes of biomass, CA and CephC under different oxygen uptake for all eighteen combinations of the objective function (see also supplementary material 1).

**Table 3:** Metabolic scenarios for all objective functions evaluated.

No. Objective function	Biomass (h^−1^)				Clavulanic acid (mmol/gCDW*h)				Cephamicyn C (mmol/gCDW*h)			
	Oxygen uptake (mmol/gCDW*h)											
	2,1	4,35	9,15	14,1	2,1	4,35	9,15	14,1	2,1	4,35	9,15	14,1
1	1,433	2,156	2,848	2,848	0,000	0,000	0,000	0,000	0,000	0,000	0,000	0,000
2	1,433	2,156	2,848	2,848	0,000	0,000	0,000	0,000	0,000	0,000	0,000	0,000
3	1,433	1,917	2,848	2,848	0,000	0,000	0,000	0,000	0,000	0,205	0,000	0,000
4	1,433	1,992	2,581	2,848	0,000	0,222	1,069	2,151	0,000	0,000	0,000	0,000
5	1,433	1,992	2,581	2,848	0,000	0,222	1,069	2,151	0,000	0,000	0,000	0,000
6	1,433	1,917	2,541	2,848	0,000	0,000	0,952	2,151	0,000	0,205	0,108	0,000
7	1,433	1,992	2,581	2,848	0,000	0,222	1,069	2,151	0,000	0,000	0,000	0,000
8	1,433	1,974	2,581	2,848	0,000	0,196	1,069	2,151	0,000	0,000	0,000	0,000
9	1,433	1,992	2,581	2,848	0,000	0,222	1,069	2,151	0,000	0,000	0,000	0,000
10	1,433	2,156	2,848	2,848	0,000	0,000	0,000	0,000	0,000	0,000	0,000	0,000
11	1,433	2,156	2,848	2,848	0,000	0,000	0,000	0,000	0,000	0,000	0,000	0,000
12	1,433	2,156	2,848	2,848	0,000	0,000	0,000	0,000	0,000	0,000	0,000	0,000
13	1,433	2,156	2,848	2,848	0,000	0,000	0,707	2,151	0,000	0,000	0,000	0,000
14	1,433	2,156	2,848	2,848	0,000	0,000	0,707	2,151	0,000	0,000	0,000	0,000
15	1,433	2,156	2,848	2,848	0,000	0,000	0,707	2,151	0,000	0,000	0,000	0,000
16	1,433	1,992	2,581	2,848	0,000	0,222	1,069	2,151	0,000	0,000	0,000	0,000
17	1,433	1,992	2,581	2,848	0,000	0,222	1,069	2,151	0,000	0,000	0,000	0,000
18	1,433	1,992	2,581	2,848	0,000	0,222	1,069	2,151	0,000	0,000	0,000	0,000

**Fig. 1:**
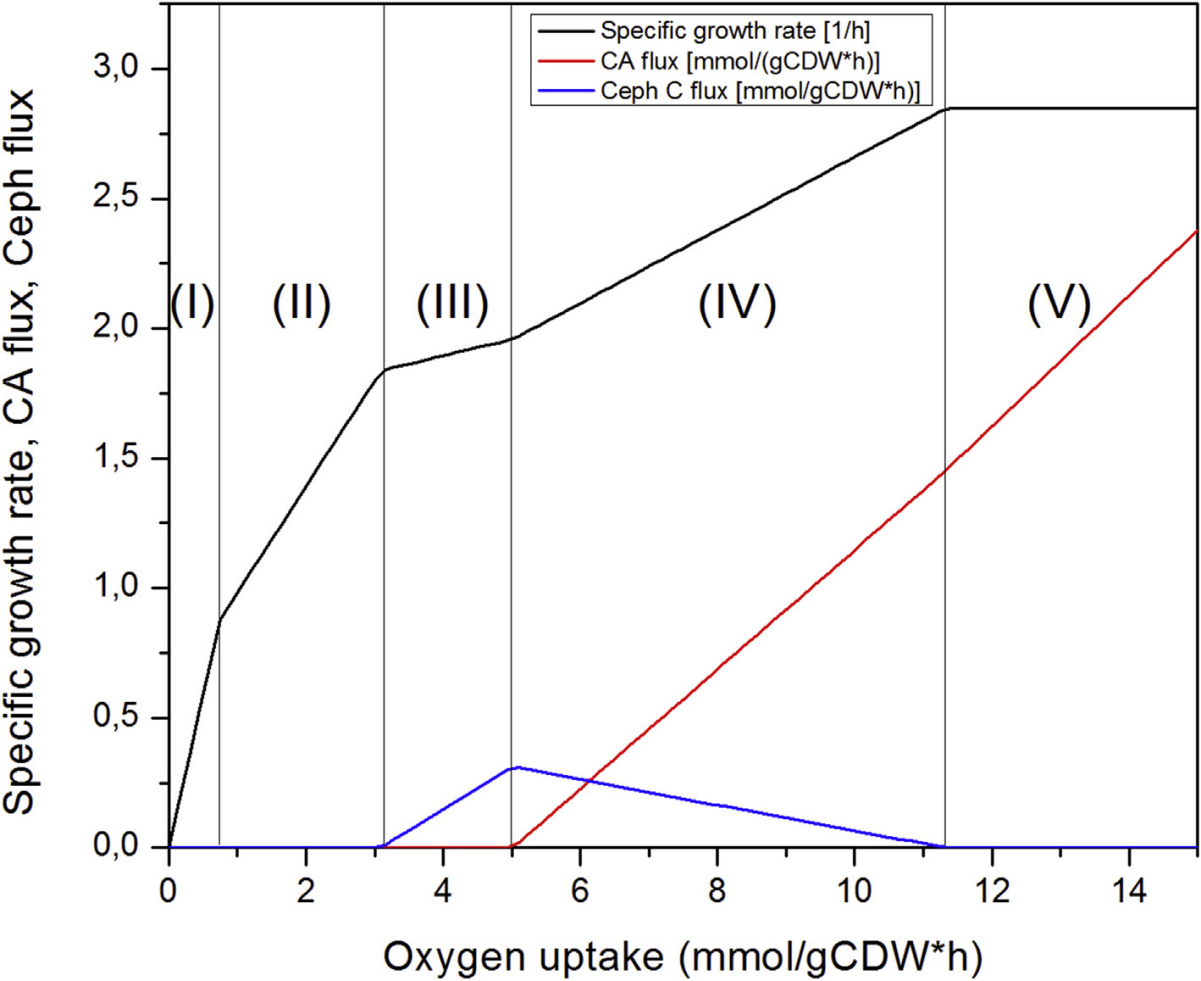
Profile of biomass, CA and CephC while varying oxygen uptake for the objective function No. 6.

Fig. 1 shows five different metabolic phenotypes of *S. clavuligerus* as a function of oxygen uptake: (I) and (II) biomass production, (III) biomass and CephC production, (IV) simultaneous production of biomass, CA and CephC and (V) production of biomass and CA. See also supplementary material 2.

## 2 Experimental design, materials, and methods

### 2.1 Model

The genome scale metabolic model reported by Ramirez-Malule et al. (2018) was used as starting point [10]. The published model consists of 1510 reactions (1305/205 internal/exchange fluxes) and 1187 metabolites (982/205 internal/external metabolites). The model was curated manually according to KEGG pathway (https://www.genome.jp/kegg/) and enzyme database (https://www.enzyme-database.org/). The improved metabolic model encompassed 1534 reactions (1322/212 internal/exchange fluxes) and 1199 metabolites (987/212 internal/external metabolites). Cytoscape was used to visualize unconnected reactions in the metabolic network [11].

### 2.2 Flux balance analysis

Flux balance analysis (FBA) was used to determine metabolic states [12], [13]. Loop law constrains was applied to all FBA simulation ensuring that infeasible loops ware not allowed [14]. The production of biomass, CA and CephC was used as objective function.

### 2.3 Optimization problem statement

Metabolic fluxes were quantified by means of a two-stage optimization approach, which is a combination of the maximization of the objective function and minimization of the overall flux [10], [15], [16]. The mathematical problem can be represented as follows:

Stage one

(1) $$maximize\;Z=\left(w_{biomass}*v_{biomass}\;+\;w_{CA}*v_{CA\;intracellular}\;+\;w_{Cephc}*v_{Cephc\;intracellular}\right)$$

subjectto:S∗v=0

$$v_{\mathrm{lb}}\leq v\leq v_{\mathrm{ub}}$$

Stage two:

(2) $$minimize\;\sum v_{i}^{2}$$

subjectto:S∗v=0

$$v_{biomass}=v_{optbiomass}$$

$$v_{CA\;extracellular}=v_{optCA\;extracellular}$$

$$v_{Cephc\;extracellular}=v_{optCephc\;extracellular}$$

$$v_{\mathrm{lb}}\leq v\leq v_{\mathrm{up}}$$

where Z is the objective function, S is the stiociometric matrix and v is the flux vector. $w_{biomass}$, $w_{CA}$ and $w_{Cephc}$ are the weighting factors for biomass, intracellular flux of CA and CephC, respectively. $v_{biomass}$, $v_{CA\;intracellular}$ and $v_{Cephc\;intracellular}$ are the biomass flux, intracellular flux of CA and CephC, respectively. $v_{optbiomass}$, $v_{optCA\;extracellular}$ and $v_{optCephc\;extracellular}$ are the optimal values for biomass and extracellular flux of CA and CephC, respectively, that resulted from solving the problem stated at stage one.

The first stage optimization problem was solved using a Gurobi solver, with a feasibility tolerance of 10^−6^, while the second stage was solved using the MATLAB's built-in *fmincon* solver, with a first order optimality and a maximum constraint violation within 10^−6^.

Different objective functions were evaluated varying the weighting factors of CA and CephC between 0, 1 y 2, whereas for the case of biomass the weight factor was varied between 1 and 2 (see Table 2).

### 2.4 Robustness analysis

A robustness analysis was carried out to evaluate the functionally of the objective function when the optimal flux of oxygen was varied [12], [13]. The identification of possible gene knockout was made by sensitivity analysis using the concept of reduced costs. The reduced cost values represent the variation of the objective functions with respect to the fluxes related to each reaction and they are represented according to the equation (3). Additionally, the shadow prices were determined following the equation (4) [13], [17].

(3) $$Z=Z_{0}+\rho ,v,\;\;\;\;\rho_{i}=-\frac{\partial Z}{\partial v_{i}}$$

(4) $$\pi_{i}=-\frac{\partial Z}{\partial b_{i}}$$

Where, $\rho_{i}$ is the reduced cost, $Z_{0}$ is the optimal solution, $v_{i}$ is an internal flux that is not in the basis solution, $\pi_{i}$ is the shadow prices and $b_{i}$ is the exchange fluxes.

### 2.5 Computational tools

COBRA Toolbox v.3.0 synchronized with Matlab^®^ as programing environment, and the Gurobi optimizer 7.5.2 was used to solve all optimization problems [18].
